# Involvement of Dendritic Cells and Th17 Cells in Induced Tertiary Lymphoid Structures in a Chronic Beryllium Disease Mouse Model

**DOI:** 10.1155/2021/8845966

**Published:** 2021-05-06

**Authors:** Alex KleinJan, Menno van Nimwegen, Karolina Leman, Ke-xin Wen, Louis Boon, Rudi W. Hendriks

**Affiliations:** ^1^Department of Pulmonary Medicine, Erasmus University Medical Center (Erasmus MC), Dr. Molewaterplein 50, 3015 GE Rotterdam, Netherlands; ^2^Polpharma Biologics Formerly Bioceros BV, Yalelaan 46, 3584 CM Utrecht, Netherlands

## Abstract

**Objective:**

To study airway pathophysiology and the role of dendritic cells (DCs) and IL-17 receptor (IL-17R) signals in a mouse model for CBD.

**Methods:**

Here, we present a CBD mouse model in which mice were exposed to beryllium during three weeks. We also exposed IL-17R-deficient mice and mice in which DCs were depleted.

**Results:**

Eight weeks after the initial beryllium exposure, an inflammatory response was detected in the lungs. Mice displayed inflammation of the lower airways that included focal dense infiltrates, granuloma-like foci, and tertiary lymphoid structure (TLS) containing T cells, B cells, and germinal centers. Alveolar cell analysis showed significantly increased numbers of CD4^+^ T cells expressing IFN*γ*, IL-17, or both cytokines. The pathogenic role of IL-17R signals was demonstrated in IL-17R-deficient mice, which had strongly reduced lung inflammation and TLS development following beryllium exposure. In CBD mice, pulmonary DC subsets including CD103^+^ conventional DCs (cDCs), CD11b^+^ cDCs, and monocyte-derived DCs (moDCs) were also prominently increased. We used diphtheria toxin receptor-mediated targeted cell ablation to conditionally deplete DCs and found that DCs are essential for the maintenance of TLS in CBD. Furthermore, the presence of antinuclear autoantibodies in the serum of CBD mice showed that CBD had characteristics of autoimmune disease.

**Conclusions:**

We generated a translational model of sarcoidosis driven by beryllium and show that DCs and IL-17R signals play a pathophysiological role in CBD development as well as in established CBD in vivo.

## 1. Introduction

Sarcoidosis is a systemic inflammatory disease of unknown cause. An inflamed lung is one of the most common features in >90% of these patients [1]. The disease is characterized by the presence of compact, organized, noncaseating granulomas that contain activated T cells, dendritic cells (DCs), macrophages, and histiocytes (epithelioid and macrophage-like cells) [2–4]. Although the disease mechanism and cause are unknown, the immunological phenotype of sarcoidosis patients indicates that the disease is multifactorial. It has been suggested that its etiology involves certain bacterial antigens, such as *Mycobacterium*, *Propionibacterium*, and Mkat G [5–7]. Accordingly, animal models based on these antigens show activation of various immune cells including T and B cells and DCs.

In human pulmonary sarcoidosis, we and others observed the increased presence of T helper 17.1 (Th17.1) cells, which have the capacity to express both IL-17 and IFN*γ* in both bronchoalveolar lavage (BAL) and mesenteric lymph node (MLN) [8–12]. The contribution of the Th17 cell lineage to the pathophysiology of sarcoidosis is also supported by our recent identification of reduced cytotoxic T lymphocyte-associated protein 4 (CTLA4) expression on Th17 lineage cells, which may contribute to their increased activation [13]. Moreover, DCs were shown to be activated in blood and granuloma-containing tissues obtained from sarcoidosis patients, compared with controls [14]. Nevertheless, the findings of CD4^+^ T cell accumulation, oligoclonal TCR*αβ*^+^ expansions, and production of IFN*γ* and T helper (Th)1-promoting cytokines including interleukin- (IL-) 12, chemokines, and chemokine receptors at sites of inflammation provide evidence for a pathological antigen-driven Th1 response [15, 16].

In the context of influenza, it became clear that pulmonary DCs act locally by modifying the pulmonary environment in such a way that they facilitate local immune responses and thereby are important for the maintenance and function of inducible bronchus-associated lymphoid tissue (iBALT) [17, 18]. DCs and the proinflammatory cytokine IL-17 are suggested to participate in and contribute to granuloma formation and cell fusion [19], resulting in the frequently observed formation of multinucleated (giant) cells. In humans living in regions with high prevalence of *Mycobacterium tuberculosis* infection, peripheral blood contains high frequencies of IL-17^+^ and IL-22^+^ memory T helper cells, which may have protective properties against tuberculosis [20]. In mouse models and in humans with active pulmonary tuberculosis, both IL-17- and IL-22-producing CD4^+^ T cells and IL-17^+^*γδ* T cells were shown to contribute to the antimycobacterial immune response [21, 22]. In addition to IL-17, also chemokines such as CXCL10 (binding to CXCR3) and CXCL13, as well as IL-23, are reported to be important for the formation of tertiary lymphoid structures (TLS) [21, 23, 24].

Over the past decades, pathological beryllium (Be) metal exposure has frequently been observed. Patients sensitive to inhaled Be showed a clinical picture similar to pulmonary sarcoidosis [25–27]. Chronic beryllium disease (CBD) is a lifelong CD4^+^ T cell-mediated lung inflammatory illness, common among certain industrial workers who handle beryllium [28]. Mice can be exposed to beryllium in such a way that they develop CBD-like pulmonary sarcoidosis with similarity to human CBD [29–32]. It has been reported that Be exposure can serve as adjuvants promoting DC activation and inducing a structural change in HLA-DP2. Hereby, neoantigens are generated that lead to immune activation with similarity to autoimmunity, which might be the basis for disease pathology [33].

In this study, we established a Be exposure protocol to generate an *in vivo* mouse model for CBD. In this model, we investigated the pathophysiological role of DCs and IL-17 in CBD development as well as in established CBD *in vivo*. In particular, we explored the involvement of DCs and IL-17 in the formation of granulomas and TLSs.

## 2. Material and Methods

### 2.1. Mice

Six-week-old mice (C3H/HeN and C57bl/6) were purchased from Envigo (Zeist, The Netherlands). CD11c-diphtheria toxin receptor (DTR) transgenic mice (BALB/c) [34] were crossed with C3H wild-type (WT) mice. BALB/c x C3H (F1) mice were used in experiments investigating DC dependency. IL-17R KO mice (C57bl/6) have been previously described [35]. Mice were housed under SPF conditions and provided with food and water *ad libitum*. All experiments were approved by the animal ethics committee at Erasmus MC, Rotterdam.

### 2.2. Induction of CBD

C3H/Hej or C57bl/6 mice were exposed to Be metal, beryllium oxide (BeO), and aluminum Be (AlBe) in 80 *μ*l suspension (180 *μ*g, unless indicated otherwise) by intratracheal injection under isoflurane anesthesia for three days per week during three weeks. Mice were sacrificed at 8, 16, 24, and 32 weeks after initial exposure.

BALB/c x C3H (F1) CD11c-DTR and WT littermate control mice were treated with Be and BeO to induce CBD. Diphtheria toxin (DT) was administered intratracheally to 10-week-old mice. Mice were sacrificed two days after DT administration. IL-17R KO and WT C57bl/6 control mice were exposed to Be and BeO to induce CBD. Mice were sacrificed 16 weeks after initial exposure.

### 2.3. Tissue Preparation

Mice were sacrificed and bled, and bronchoalveolar lavage (BAL) was performed by flushing the lungs 3 times with 1 ml PBS containing ethylenediaminetetraacetic acid (EDTA, Sigma-Aldrich), using standard procedures. The lungs were inflated with Tissue-Tek O.C.T. compound 1 : 1 in PBS and frozen in liquid nitrogen and stored by -80°C until use [36].

### 2.4. Flow Cytometric Analysis

BAL fluid, lung, and mediastinal lymph node (MLN) cells were collected for cellular differentiation by flow cytometry as previously described (Supplementary Table (available here)) [37]. Intracellular flow cytometric analysis of cytokines was performed as previously described. Fixable Aqua Life/Dead for 405 nm (Invitrogen, Molecular Probes) was used to distinguish between live and dead cells [36]. Cells were analysed using a LSR II flow cytometer (BD Biosciences) and FlowJo software (Tree Star Inc., USA).

### 2.5. Cytokine Detection by ELISA

Cytokine detection in BAL or cell culture supernatant was performed by enzyme-linked immunosorbent assay (ELISA) according to the protocol of the Ready-Set-Go (eBioscience) or OptEIA (BD) kit.

### 2.6. Immunohistochemistry

6 *μ*m frozen lung sections were stained with monoclonal antibodies specific for either GR1 (clone RB6-8C5), CD3 (145-2C11), CD4 (L3T4), CD8 (Ly-2), CD11c (N418), GL7, IgD (11-26c), F4/80 (BM8), and B220 (RA3-6B2), followed by alkaline phosphatase- (AP-) conjugated or horse radish peroxidase- (HRP-) conjugated secondary antibodies, using New Fuchsine and Nova Red as substrate, respectively. Nuclei were stained blue with Gill's hematoxylin. Double stainings were performed by combining AP and HRP staining protocols with the combined addition of the primary antibodies and subsequently the secondary antibodies together. AP detection by Fast Blue was followed by HRP detection by Nova Red [34].

For the quantification of organized focal dense infiltrates, we included immune cell accumulations with a minimal size of 15 cells per lung section with a minimum size of 0.5 cm^2^. In these infiltrates, we scored the presence or absence of a clear B cell zone or a clear T cell zone.

### 2.7. Detection of Autoantibodies

Serum samples (diluted 1/100 in PBS) or BAL fluid samples (undiluted) were incubated on Kallestad HEp-2 slides (Bio-Rad Laboratories) for 1 hour. As a secondary antibody, Alexa Fluor 488-conjugated donkey anti-mouse IgG (Invitrogen) was applied. After embedding the HEp2 slides in glycerol (Sigma-Aldrich), fluorescence intensity was evaluated using a LSM 510 META confocal fluorescence microscope (Zeiss) and LSM Image Browser Version 4.2.0.12 software (Zeiss).

### 2.8. Statistical Analysis

Statistical analyses were performed with SPSS (SPSS Inc., Chicago, IL), using the Kruskal-Wallis test followed by a Mann-Whitney *U* test. *p* values < 0.05 were considered significant. Tests that did not reach significance (*p* > 0.05) are not indicated in the figures.

## 3. Results

### 3.1. Mice Develop CBD Pathology 8 Weeks after Initial Beryllium Exposure

Female C3H mice were subjected to several treatment strategies, regarding dose (18 *μ*g or 180 *μ*g) and exposure to different beryllium compounds (Be, BeO, and AlBe) invariably three times weekly for 3 weeks. Be particles were applied i.t. in an emulsion in PBS in a volume of 80 *μ*l. Mice were analysed at various time points after exposure. These treatment regimens were well tolerated, as indicated by similar weight gains of the exposed mice compared with PBS-treated control mice over a period of 8 weeks (Supplementary Figure 1). As early as ~6 weeks after the initial exposure to 180 *μ*g Be or BeO, a fraction of mice showed pathophysiological abnormalities in the lung, which were characterized by focal dense infiltrates and small granulomas. More pronounced, significant abnormalities were observed at ~8 weeks after the start of exposure. Exposure to AlBe or low doses of Be or BeO (18 *μ*g) resulted in only mild, nonsignificant lung pathology, based on analysis of H&E stainings (data not shown).

**Figure 1:**
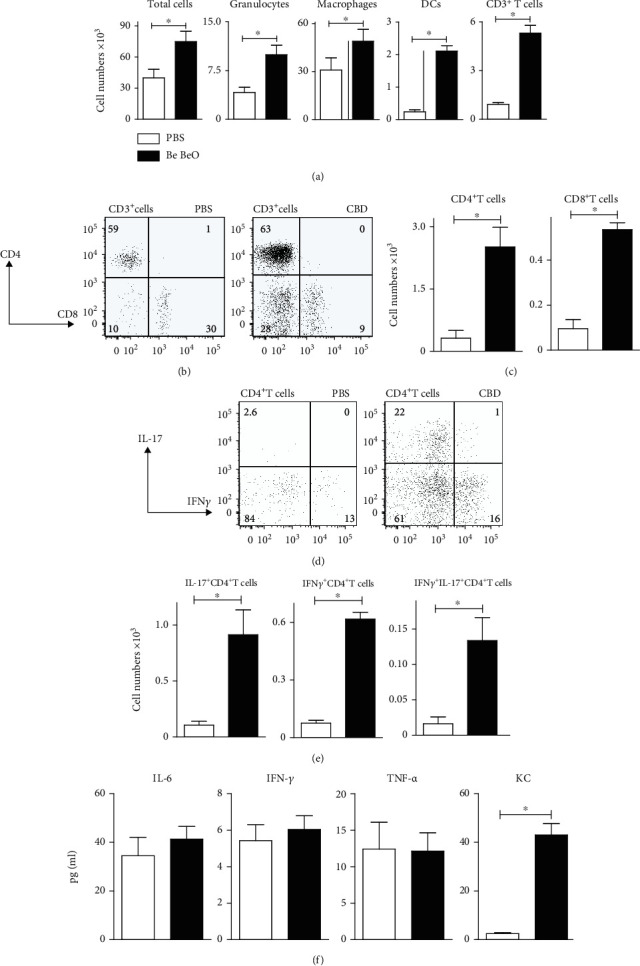
Characterization of the inflammation in BAL fluid of CBD mice. (a) Quantification of FACS analysis of the indicated cell populations in BAL at 8 weeks after initial exposure (granulocytes (GR1^+^ cells), macrophages (autofluorescence and macrophage gate), DCs (CD11c^+^MHCII^+^), and CD3^+^ T cells). (b–e) Intracellular flow cytometry analysis of cytokine production of BAL cells that were restimulated with PMA and ionomycin in the presence of Golgi-stop. CD3^+^ T cells were gated and analysed for their CD4/CD8 profile (b), which was quantified (c). CD3^+^CD4^+^ T cells were gated and analysed for intracellular cytokines (d), which were quantified (e). (f) Cytokine levels in BAL fluid, as determined by ELISA. Mice (*n* = 5 − 7 per group) were treated with PBS (controls, white bars) or Be/BeO (black bars). Mann-Whitney *U* test: ^∗^*p* < 0.05. Data are representative for 2 experiments performed.

For subsequent experiments, C3H or C57bl/6 mice were exposed to beryllium using a 1 : 1 mixture of Be and BeO (180 *μ*g in 80 *μ*l), 3 times weekly for 3 weeks. At time points < 8 weeks after the initial exposure, no significant abnormalities were observed, as measured by flow cytometry (Supplementary Figure 2; data not shown). From ~8 weeks after the first exposure onwards, Be/BeO-exposed mice manifested various airway abnormalities, which we marked as CBD. BAL cells—representing the alveolar compartment—were characterized and quantified by flow cytometry (Figure 1; see Supplementary Figure 3 for gating strategy). Total numbers of BAL cells, consisting of GR1^+^ neutrophils, macrophages, DCs, and CD3^+^ T cells, were significantly higher in CBD than in PBS-treated mice (Figure 1(a)). These abnormalities were observed up to 32 weeks (last evaluated time point) after the start of exposure.

From these findings, we conclude that we established an *in vivo* mouse model for CBD with involvement of innate and adaptive immune cells.

### 3.2. CBD Mice Display Lymphocytosis of IL-17-Producing CD4^+^ T Cells in BAL

When we quantified the numbers of CD4^+^ and CD8^+^ T cells in the BAL of CBD mice at ~8 weeks after the first exposure to Be/BeO, we found that both populations were significantly increased (Figures 1(b) and 1(c)). Interestingly, CBD mice displayed increased CD4 : CD8 ratios thereby paralleling the CD4^+^ T cell lymphocytosis in the BAL of sarcoidosis patients [1].

Next, we explored which T cell cytokines were produced in the airways in CBD mice. BAL cells were analysed by intracellular cytokine staining after 4 hours of stimulation with PMA and ionomycin (Figure 1(d)). BAL CD4^+^ T cells producing IL-17, IFN*γ*, or both cytokines (reflecting Th17.1 cells) were significantly increased in CBD mice, compared with PBS control mice, both in proportions and in absolute numbers (Figures 1(d) and 1(e)). Quantification of cytokines in BAL showed that IL-6, IFN*γ*, and TNF-*α* levels were not different between PBS and CBD mice, but levels of KC (IL-8) in BAL fluid of CBD mice were significantly increased, compared with PBS control mice (Figure 1(f)).

These observations show that the CBD phenotype in mice is accompanied by a significantly increased production of IL-17 by CD4^+^ T cells, as well as the presence of neutrophil chemoattractant cytokine KC in the alveolar compartment.

### 3.3. Abundant Presence of Inflammatory Cells in BAL and Lung Tissue in CBD Mice

**Figure 2:**
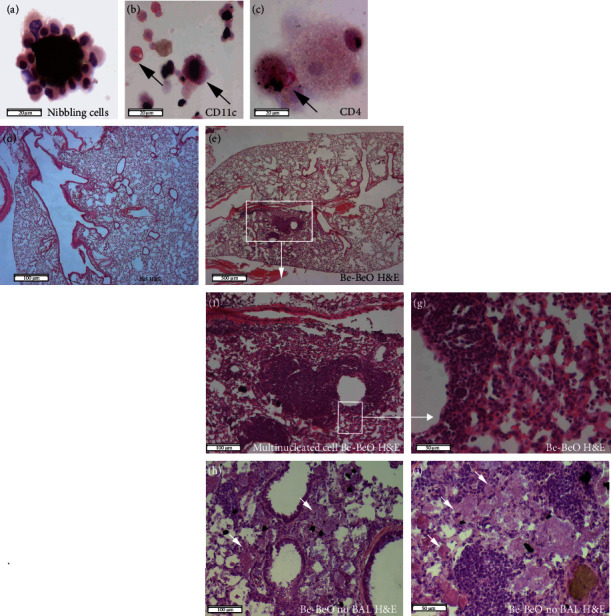
Identification of nibbling cells around beryllium particles and formation of focal dens infiltrates. (a–c) Cytospin analysis of BAL cells from CBD mice, analysed by hematoxylin and eosin (H&E) staining 8 weeks after initial exposure (a); immunohistochemical staining for CD11c^+^ alveolar macrophages or DCs (red) (b); and for CD4^+^ T cells (red) (c). (d–i) Hematoxylin and eosin (H&E) analysis of lung sections. Staining of PBS-treated mice (d); Be-BeO-treated CBD mice following BAL collection (e–g) or without prior BAL collection, showing histiocytic and focal dense infiltrates. Black dots are Be particles, and brown gray dots are BeO particles. Arrows indicate the macrophage histiocytes, which were not present when BAL was performed. Data are representative for 3-5 mice analysed in each group. Data are expressed as the means ± SEM. Bars are 20 *μ*m (a, b, c), 50 *μ*m (f), 100 *μ*m (h), and 500 *μ*m (d, e).

Next, we performed histocytologic analyses of the BAL cells and lungs of CBD mice at ~8 weeks after initial beryllium exposure. First, we observed the presence of Be and BeO particles surrounded by nibbling cells (Figure 2(a)) in BAL cell suspensions. Cytochemical analysis of BAL cells confirmed the presence of CD11c^+^ cells (macrophages and DCs) (Figure 2(b)) and CD4^+^ T cells (Figure 2(c)). Whereas histological characterization of formalin-fixed, paraffin-embedded lung tissues showed no abnormalities in PBS control mice (Figure 2(d)), mice (C57bl/6 or C3H) exposed to a mixture of Be/BeO showed peribronchial, perivascular, and parenchymal focal dense infiltrates at ~8 weeks after initial exposure (Figures 2(e)–2(g)). In lung tissue obtained from CBD mice without prior BAL collection, the alveolar space was filled with large cells with macrophage morphology, which formed granulomas and were surrounded by focal dense infiltrates (Figures 2(h) and 2(i)).

In summary, CBD mice showed enhanced lung inflammation with the characteristics of focal dense infiltrates and granulomas.

### 3.4. Characterization of CBD Cellular Infiltrates: CD11c^+^ DCs and Monocytes

**Figure 3:**
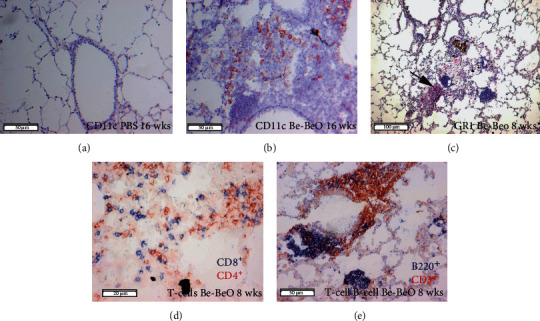
Characterization of inflammatory structures in the CBD lungs. Immunohistochemistry of lung sections, together with hematoxylin and eosin (H&E) staining. (a) Limited numbers of CD11c^+^ cells in the lungs of PBS control mice. (b) Abundant presence of CD11c^+^ cells forming granuloma-like structures in CBD mice 16 weeks after initial exposure Be BeO (c) Gr-1^+^ granulocytic inflammation in CBD mice 8 weeks after initial exposure. (d) Diffuse and dense infiltration of CD4^+^ (red) and CD8^+^ (blue) T cells. (e) Perivascular focal dense infiltration, reflecting TLS, consisting of B220^+^ B cells (blue) and CD3^+^ T cells (red) in the lungs of CBD mice at 8 weeks. Data are representative for 5-7 mice analysed in each group. Bars are 20 *μ*m (d), 50 *μ*m (a, b, e), or 100 *μ*m (c).

Next, we characterized the observed clusters of inflammatory cells in more detail and identified the presence of CD11c^+^ cells (DCs or alveolar macrophages) and GR1^+^ granulocytes; both of which were very low in PBS control mice (Figure 3(a)) and abundantly present in CBD mice (Figures 3(b) and 3(c)). At 8 weeks after the first exposure, CD8^+^ T cells were mainly presently dispersed in the lung parenchyma, whereas the majority of CD4^+^ T cells were present in aggregates of inflammatory cells around the large airways, forming inducible TLS (Figure 3(d)).  Figure 3(e) shows a representative example of a TLS stained for B220^+^ B cells and CD3^+^ T cells. We detected cell clusters indicating granuloma formation in CBD mice from ~8 weeks up to ~32 weeks after initial Be exposure, but not in PBS-treated mice.

### 3.5. Characterization of Myeloid Populations in BAL of CBD Mice

In the lung, close proximity of DCs and CD4^+^ T cells was seen in areas of inflammation and granuloma-like structures, suggesting that DCs locally stimulated antigen-specific primed T cells. Moreover, stainings for CD11c (Figure 3(b)) indicated that DCs or alveolar macrophages contributed to granuloma formation in CBD, thereby paralleling the finding of CD11c^+^ cells in human granulomas of sarcoidosis patients [14].

**Figure 4:**
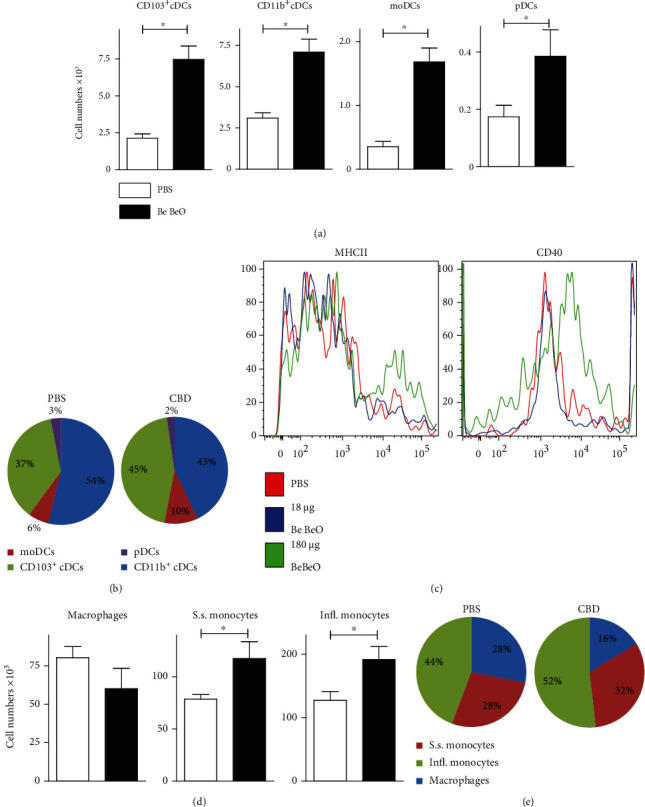
DC subset analysis in BAL fluid of CBD mice. (a) Quantification of DC subset numbers in BAL of PBS-treated mice (white bars) and CBD mice (black bars) 8 weeks after initial exposure. (b) Pie charts showing proportions of DC subsets in BAL fluid of PBS-treated and CBD mice. (c) DC activation as measured by flow cytometry staining of surface expression of MHCII and CD40 on gated CD11c^+^ cells from the nonautofluorescent fraction. (d) Quantification of the indicated macrophage and monocyte fractions in BAL of PBS-treated mice (white bars) and CBD mice (black bars) (steady-state (S.s.) monocyte; inflammatory (Infl.) monocytes). Pie charts showing proportions of macrophage/monocytes in BAL fluid of PBS-treated and CBD mice. Data are expressed as the means ± SEM (a, d) (Mann-Whitney *U* test: ^∗^*p* < 0.05). Data are from *n* = 4 − 5 mice per group and representative for 2 experiments.

As shown in Figure 1(a), Be-treated mice showed elevated numbers of DCs in BAL fluid. When we characterized individual lung DC subsets, including CD103^+^ DCs, CD11b^+^ DCs, monocyte-derived DCs (moDCs), and plasmacytoid DCs (pDCs) [38], we found that the numbers of all four subsets were increased in the BAL of CBD mice, compared with control mice (Figures 4(a) and 4(b)). Furthermore, we found that the activation status of these BAL DCs was Be concentration-dependent: low doses of ~18 *μ*g had no effect, but upon repetitive exposure to 180 *μ*g, an increase of DC activation markers was found (shown for MHCII and CD40 in Figure 4(c)), paralleling the DC phenotype observed *in vivo* in sarcoidosis patients [8]. In contrast, alveolar macrophage numbers were not different between CBD and PBS control mice (Figure 4(d)), although the proportions of alveolar macrophages were lower in CBD mice (~16% of the total monocytes/macrophages, compared with ~28% in control mice; Figure 4(e)).

The observation that in a chronic phase the frequencies of alveolar macrophages decreased and DC numbers increased is in line with observations in *Mycobacterium tuberculosis* infection [39]. Steady-state and inflammatory CD11b^+^CD11c^−^ monocytes, characterized as MHCII^−^Ly6C^+^ and CD64^−^Ly6C^−^, respectively, were significantly higher in number in CBD mice, compared to PBS control mice (Figures 4(d) and 4(e)).

In conclusion, these data show that CBD in mice is associated with DC accumulation and activation, as indicated by CD11b expression, as well as monocyte accumulation. However, we did not find evidence for the involvement of alveolar macrophages.

### 3.6. Characterization of CBD Cellular Infiltrates: B Cells and TLSs

Immune responses to respiratory infections as well as local pathology in chronic inflammatory lung diseases [17, 18] are associated with TLS formation. TLSs consist of segregated B and T cell areas, lymphatic vessels, and high endothelial venules. After inflammation is resolved, TLS is maintained for months, independently of inflammation [17, 40], and consists mainly of B cells and small populations of T cells.

**Figure 5:**
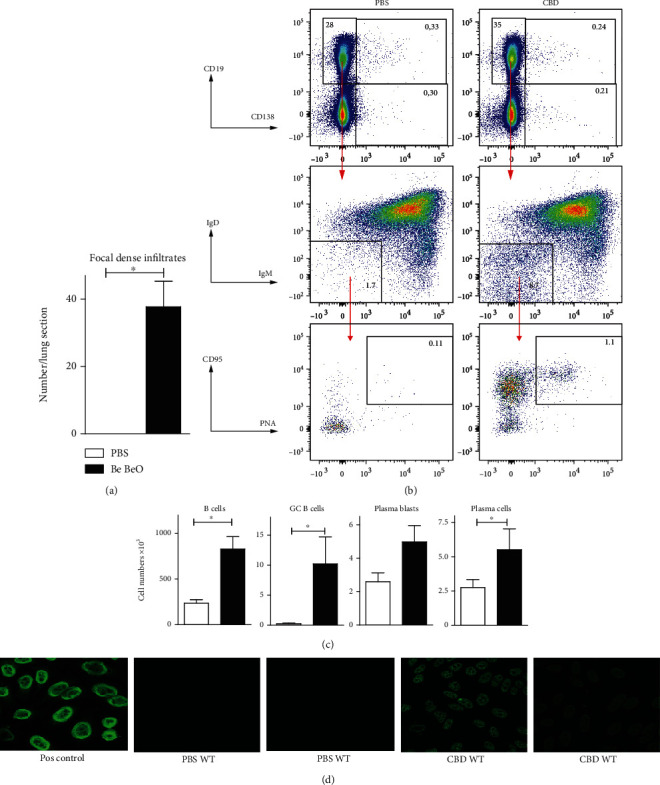
Characterization of B cell involvement in CBD mice. (a) Quantification of the numbers of focal dense infiltrates in lung sections, normalized for area in PBS-treated mice (white bar) and CBD mice (black bar) 8 weeks after initial exposure. (b) Flow cytometry analysis of B cells in single-cell lung digests of PBS mice (left) and CBD mice (right). CD19-positive cells are gated and analysed for IgM, IgD, PNA, and CD95. (c) Quantification of total B cells, CD95^+^PNA^+^ B cells, CD19^+^CD138^+^ plasma blasts, and CD19-CD138^+^ plasma cells in single-cell lung digest of PBS controls (white bars) and CBD mice (black bars). (d) Serums obtained from the indicated mice were assayed on Hep2 slides to detect IgG autoantibodies; serums from MRL-lpr mice served as a positive control. The results shown were expressed as the means ± SEM ((a) and (c); Mann-Whitney *U* test: ^∗^*p* < 0.05). The results shown represent one out of two independent experiments with 3-4 mice per group.

In CBD mice, we first quantified the numbers of focal dense infiltrates per lung section and observed significant numbers of these lymphoid-like structures, containing B cells and T cells in an organized matter (Figure 5(a)). Next, we used flow cytometry to characterize the B cell compartment in lung single-cell suspensions obtained after enzymatic digestion with liberase of the left lung lobe from CBD mice and PBS control mice (Figure 5(b)). We found that the numbers of total CD19^+^ B220^+^ B cells, IgM^−^IgD^−^CD95^+^PNA^+^ CD19^+^ germinal center (GC) B cells, CD19^+^CD138^+^ plasma blasts, and CD19^−^CD138^+^ plasma cells were substantially increased in CBD mice, compared with PBS control mice (Figure 5(c)).

The presence of TLSs in the context of autoimmune disease is often accompanied with autoantibodies [41, 42]. To detect IgG autoantibodies, we investigated the serum obtained from C57bl/6 CBD and PBS control mice by analysis of Hep2 slides. We observed antinuclear IgG autoantibodies in all 7 serum samples from CBD mice, whereas in none of the PBS control mice, IgG autoantibodies were detected (Figure 5(d)). The CBD sera showed a wide range of different staining patterns, ranging from pleomorphic nuclear staining, coarse speckled nucleoplasmic staining to homogenous nucleoplasmic staining with negative nucleoli, whereby no predominant staining pattern was seen. In BAL fluid, no autoantibodies for either IgM or IgG were detectable using Hep 2 slides.

Thus, our findings provide evidence for the formation of organized lymphoid tissue in CBD mice. Particularly, the observation of local GC B cell and plasma cell formation confirmed the presence of active organized TLSs. The presence of inducible TLSs, together with autoantibodies, points at an important parallel between CBD and autoimmune disease.

### 3.7. Depletion of CD11c^+^ Cells Eradicates CBD Inflammation

It has been reported that DCs are crucial for maintenance of established TLSs in the lung of influenza virus-infected mice [17, 18]. We wanted to address the pathophysiological role of CD11c^+^ cells in CBD and therefore crossed C3H/Hej mice with CD11c-DTR transgenic BALB/c mice [43, 44]. DCs in CD11c-DTR mice can be efficiently depleted in the lung by i.t. DT treatment. CD11c-DTR C3H/Hej x BALB/c (F1) mice were treated with Be/BeO or PBS as a control. CBD could be induced in these F1 mice similar to WT C3H/Hej mice (data not shown). Within 24 hours after i.t. injection of 50 ng DT in naïve CD11c-DTR F1 mice, we observed local efficient depletion of CD11c^+^MHCII^+^ DCs and alveolar macrophages in the lung and MLNs (see Supplementary Figure 4) [43, 44].

**Figure 6:**
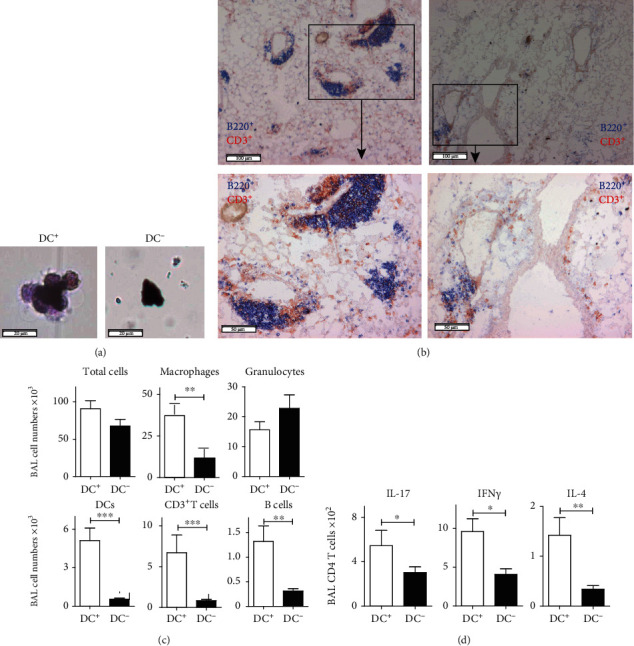
DCs are critical for maintenance of TLSs in the lung of CBD mice. (a) Cytospin analysis of BAL fluid CBD mice, analysed by hematoxylin and eosin (H&E) staining, showing that nibbling cells around beryllium particles were seen in mice in which DCs were present (DC^+^), but not in mice in which DCs were depleted using the CD11c-DTR system (DC^−^) 8 weeks after initial exposure (bar, 20 *μ*m). (b) Lung tissue sections showing the presence of TLSs with perivascular and peribronchial infiltrates (DC^+^, left), but only residual cells are seen as small diffuse infiltrates in lung tissue from DC-depleted mice (DC^−^, right) (bar upper panel 100 *μ*m; lower panel 50 *μ*m). (c, d) Quantification of cellular inflammation of the indicated cell types (c) or cytokine production by CD4^+^ T cells (d) in BAL fluid from DC^+^ (white bars) and DC-depleted (DC^−^, black bars) CBD mice. The results shown are expressed as the means ± SEM and represent one out of two independent experiments with 5-6 mice per group. Mann-Whitney *U* test: ^∗^*p* < 0.05, ^∗∗^*p* < 0.01, and ^∗∗∗^*p* < 0.001.

CD11c-DTR and control CBD mice were treated with an i.t. injection of 50 ng DT and analysed 2 days after DT treatment. In CD11c-DTR CBD mice, but not in control CBD mice, the depletion of CD11c^+^ cells resulted in the absence of nibbling cells around Be particles (Figure 6(a)) and resolution of TLS (Figure 6(b)). Quantification of BAL cells demonstrated a significant reduction of CD11c^+^MHCII^+^ DCs, CD3^+^ T cells and CD19^+^ B cells, and macrophages, whereas granulocytes (GR1^+^cells) showed no changes (Figure 6(c)). Additional controls, including i.t. PBS-injected CD11c-DTR and non-Tg CBD mice, had high amounts of CD11c^+^ cells in the BAL (data not shown). DC depletion was also associated with a significant reduction in the total numbers of IL-17, IFN*γ*, or IL-4 cytokine-producing CD4^+^ T cells (Figure 6(d)) in BAL, due to a reduction of the total numbers of CD3^+^ T cells present in the BAL of CBD mice (Figure 6(c)).

Collectively, from these experiments, we conclude that DCs are essential in CBD to maintain the focal dense infiltrates containing cytokine-producing T cells.

### 3.8. IL-17R Signaling Plays a Crucial Role in Granuloma Formation in CBD Mice

**Figure 7:**
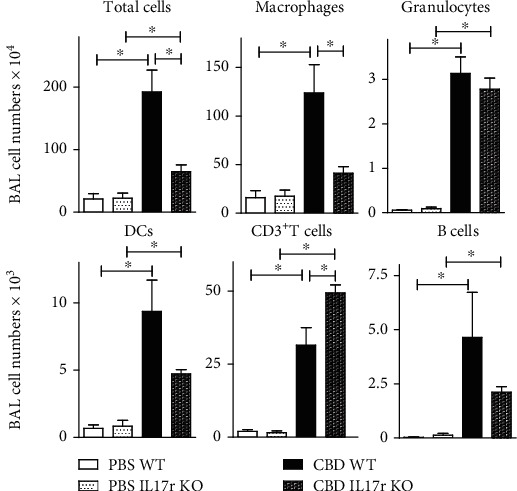
IL-17R signaling is important for CBD inflammation. Quantification of flow cytometric analyses of the indicated populations of BAL cells in four groups of mice 8 weeks after initial exposure. The results shown are expressed as the means ± SEM and represent one out of two independent experiments with 3-4 mice per group. Mann-Whitney *U* test: ^∗^*p* < 0.05.

The proinflammatory cytokine IL-17 has been implicated in the pathogenesis of various granulomatous diseases [8–11, 13, 45] in particular in the formation of mycobacterial infection-induced granulomas in the lung and in sarcoidosis [8, 20, 45, 46]. Since IL-17 is produced abundantly in CBD (Figure 1(e)), we investigated the contribution of the IL-17 receptor (IL-17R) to CBD pathology. To this end, congenic IL-17R KO mice and WT C57bl/6 control mice were exposed to Be/BeO or PBS as a control. Sixteen weeks after initial Be treatment, WT CBD mice showed robust inflammation in BAL fluid, as evidenced by increased numbers of total cells, macrophages, DCs, T cells, and B cells, compared with PBS control mice (Figure 7). The inflammation in IL-17R-deficient CBD mice was less strong when compared to CBD WT mice, as indicated by the presence of only a modest increase of total cells and macrophages in the BAL (Figure 7). Whereas the numbers of DCs and B cells tended to be lower in the IL-17R-deficient CBD mice, the numbers of T cells in the BAL were significantly higher, compared to WT CBD mice. No differences were found between IL-17R-deficient and WT PBS control mice.

The absolute numbers of CD4^+^ T cells producing IL-17, IFN*γ*, IL-4, or IL-13 were significantly higher in BAL fluid from CBD mice than in PBS control mice (Supplementary Figure 5). Both the absolute numbers and the proportions (data not shown) of IL-17-producing CD4^+^ T cells were significantly increased in IL-17R KO CBD mice, compared to WT CPD mice. It is conceivable that the proportions of IL-17^+^ CD4^+^ T cells were increased in IL-17R KO CBD mice, because of a defective autofeedback loop [47], supporting the notion that the IL-17R is important in clearance of IL-17 from the lung [47].

**Figure 8:**
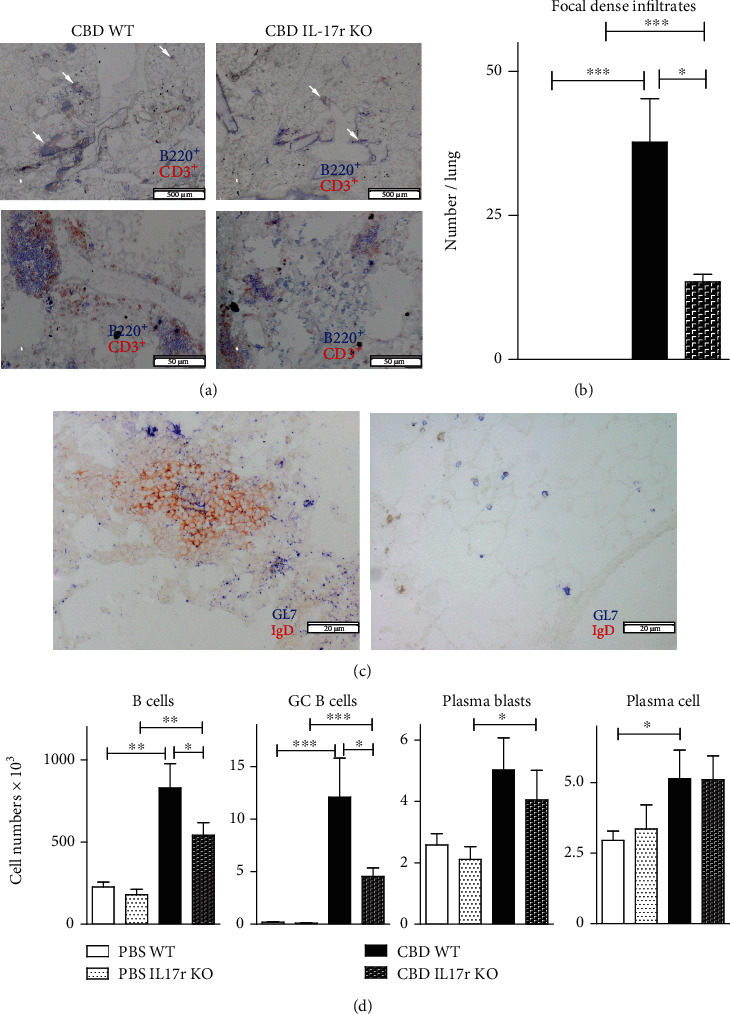
IL-17 signaling is important for TLS formation in CBD pathology. (a) Immunohistochemical analysis of lung tissue sections stained for B220^+^ B cells (blue) and CD3^+^ T cells (red) of the lungs obtained from CBD WT and CBD IL-17R KO mice, as indicated, at 8 weeks after Be/BeO exposure. Arrows indicate perivascular focal dense infiltrates, reflecting TLSs of the lungs of CBD mice. Scale bars: 500 *μ*m (upper panels) and 50 *μ*m (lower panels). (b) Quantification of focal dense infiltrates in lung sections of the indicated mice. (c) Immunohistochemical analysis of a lung tissue section for IgD (red) and the GC marker GL7 (blue) from a WT CBD mouse, conforming the presence of a GC in the TLS 20 *μ*m. (d) Quantification of flow cytometric analyses of the indicated B-lineage populations of single-cell lung digest (d). The results shown are expressed as the means ± SEM and represent one out of two independent experiments with 4-7 mice per group. Mann-Whitney *U* test: ^∗^*p* < 0.05, ^∗∗^*p* < 0.01, and ^∗∗∗^*p* < 0.001.

Histological analysis of the lungs showed granuloma structures and focal dense infiltrates (CD3^+^ and B220^+^), which can be identified as TLS because of the presence of a certain extent of organization (Figure 8(a)). Quantification of the numbers of focal dense infiltrates demonstrated more and larger structures in the CBD WT mouse lungs, compared with the CBD IL-17R KO mouse lungs (Figure 8(b)). PBS control mice showed no focal dense or diffuse infiltration in the lungs. To support the presence of lymphoid structure organization, double stainings for IgD and the GC B cell marker GL7 were performed. Often GL7 positivity was seen associated with IgD^+^ areas suggesting the presence of GCs (Figure 8(c)) which were hardly detectable in CBD IL-17R KO mice.

Next, we analysed digested lung single-cell suspensions for the presence of total B cells, GC B cells, plasma blast, and plasma cells. The numbers of total CD19^+^ B cells and GC B cells were higher in CBD mice than in PBS control mice, both for WT and IL-17R KO mice (Figure 8(d); data not shown). For plasma blasts and plasma cells, similar differences were seen, albeit partly significant (Figure 8(d)). Importantly, the induction of total B cells and GC B cells in CBD was significantly reduced in IL-17R KO mice, compared with WT mice.

In summary, these findings show that the IL-17R signaling plays an important supportive pathophysiological role in beryllium-induced TLS formation.

## 4. Discussion

CBD in humans is a sarcoidosis-like disease with a known causative antigen, beryllium. In our model, we identified a set of disease hallmarks: (i) an increase of IL-17^+^CD4^+^ T cells in the BAL fluid; (ii) activation of DCs in the BAL fluid; (iii) the presence of focal dense infiltrates, i.e., granuloma-like structures, in the lungs; (iv) the presence of TLS in the lung, containing GC B cells; and (v) circulating antinuclear autoantibodies.

Beryllium exposure in animal models has previously been employed to investigate its toxicity and to study sarcoidosis-like disease [29, 31]. To the best of our knowledge, however, this is the first time that it is shown that beryllium can induce TLSs, IL-17-producing Th17 cells, and IL-17/IFN*γ* double-producing Th17.1 cells, paralleling findings in sarcoidosis patients [8]. Th17 cells, including Th17.1 cells, which have the capacity to produce IFN*γ* next to IL-17 or IFN*γ* alone, are associated with various autoimmune diseases and sarcoidosis [10, 35, 48, 49]. Here, we provided evidence that IL-17R signaling is an important contributor of CBD: when we blocked IL-17R signaling, using IL-17R-deficient mice, pathology was reduced. It has been reported that both in CBD mouse models and in sarcoidosis patients, pulmonary *in vivo* DC activation is observed [14, 16, 33]. Here, we demonstrated that when DCs were depleted—at a disease stage in which complete TLSs were formed—these structures resolved and the numbers of T and B cells dropped in the alveolar space. These findings show that besides viral and bacterial infections or chronic autoimmune disease [50], also exposure to metal, including beryllium, can be responsible for the induction of TLSs in the lung.

In our CBD model, there was considerable heterogeneity in the pulmonary infiltrates. These included fibrotic structures containing CD11c^+^ cells at one side of the spectrum and TLSs containing separated B and T cell zones on the other side, whereby both structures were seen in the same lung. IL-17 has previously been implicated in various conditions characterized by granuloma formation [8, 19]. Our data showed that IL-17 plays a supportive role in the formation of these inflammatory structures, because we observed less and smaller TLS in IL-17R KO CBD mice compared to WT CBD mice. At the same time, our findings indicate that the IL-17R pathway is not the only factor critically involved in CBD pathology, since there is still remaining pathology in the absence of IL-17R signaling. Therefore, additional factors will play a role in beryllium-driven disease pathogenesis.

In a *Mycobacterium bovis* infection model, IL-17 expression was detected early after pulmonary infection and IL-17-deficient mice showed impaired granuloma formation [51]. In humans living in regions with high prevalence of *Mycobacterium tuberculosis* infection, peripheral blood contains high frequencies of IL-17^+^ and IL-22^+^ memory T helper cells, which may have protective properties [20]. In mouse models and in humans with active pulmonary tuberculosis, both IL-17- and IL-22-producing CD4^+^ T cells as well as IL-17^+^*γδ* T cells were shown to contribute to the antimycobacterial immune response [21, 22]. Lung injury in a mouse model for chronic granulomatous disease with lethal aspergillosis was shown to involve unrestrained *γδ* T cell reactivity and dominant production of IL-17 [52]. In Langerhans cell histiocytosis, which is accompanied by aggressive chronic granuloma formation, yet another cell population, DCs, was shown to synthesize IL-17 [19, 23]). An IL-17-dependent pathway for DC fusion was identified, which was potentiated by IFN*γ* and led to giant cell formation. In this context, interesting parallels between Langerhans cell histiocytosis and sarcoidosis further include the presence of multinucleated giant cells [15, 19, 23].

It has been reported that beryllium-specific CD4^+^ T cells produce significant amounts of IFN*γ* and TNF*α* in the absence of antigen-presenting cells (APCs) [53]. Also, in our hands, *in vitro* cocultures of beryllium-pulsed bone marrow-derived DCs (mainly moDCs) with CD4^+^ T cells from CBD mice showed that beryllium presentation by DCs was not required to induce proliferation of CBD memory CD4^+^ T cells (A.K., unpublished findings). CD4^+^ T cells from MLN of CBD mice were primed for generating effector cells, because these cells proliferated more readily in PBS conditions than CD4^+^ T cells from the CBD mouse spleen did (A.K., unpublished findings). Consistent with these findings, earlier studies have shown beryllium self-presentation by BAL Th1 cells in the absence of APCs, resulting in self-activating and proliferation of Th1 cells [54]. Yet, the induction of differentiation of naïve T cells into Th17 or Th17.1 cells may be due to a subpopulation of DCs, for example, moDCs, which were also associated with induction of Th1 cell immunity [55–57], but may be in the context of CBD support Th17/Th17.1 differentiation.

DCs present in the BAL of CBD mice displayed an activated phenotype that included upregulation of MHCII and CD40 expression, which is in agreement with DC activation previously observed in asthma models [44]. CD40 expression supports GC formation [58]. Because we found that all major DC subsets were present in increased numbers in the CBD lungs, compared to controls, it is conceivable that there is not a particular DC subtype dominantly involved in CBD. Yet, depleting CD11c^+^ cells, including all DCs, abrogated granulomas and TLS, which identified a crucial role for DCs in the maintenance of granulomas and TLSs, paralleling findings in Mycobacterium and influenza infection [17, 48, 59]. TLSs in CBD were observed perivascular and peribronchial and in the parenchyma and are essentially comparable with TLSs seen in various other mouse models, including iBALT in influenza models and chronic house dust mite-driven allergic airway inflammation [60], as well as the perivascular TLSs seen in the spontaneous autoimmune model of B cell-specific overexpression of the signaling molecule Bruton's tyrosine kinase (Btk) [61]. Evidently, the differences in the TLS location are associated with the original of the stimulus, i.e., an agent applied in the lungs of WT mice, or aberrant B cell function and circulating autoantibodies. Nevertheless, the TLSs in all these models are characterized by the presence of distinct T and B cell areas with germinal centers, which we also observed in CBD (Figure 3(e) and Figure 5(c)).

Next to DCs, many other cell types are involved in various aspects of the CBD phenotype. Because we found increases in monocyte and macrophage populations, it is conceivable that also these myeloid populations are critically involved, e.g., in granuloma formation, as observed in tuberculosis [39]. Furthermore, epithelial cell damage might contribute to the disease pathology. Recently, McKee et al. observed release of IL-1*α* and DNA during the acute phase of pulmonary beryllium exposure. IL-1*α* can be seen as an important epithelial cytokine, important for epithelial cell and DC activation in response to allergens [62] and to beryllium [63].

Finally, we detected IgG autoantibodies in the serum of CBD mice, showing that beryllium exposure induces activation of autoreactive B cells. The antinuclear staining pattern found in HEp2 analysis indicates that the autoantibodies might be reactive to DNA, which is also observed in sarcoidosis patients [64]. Interestingly, it has been reported that DNA is one of the additional antigens released in the acute phase of pulmonary beryllium exposure [63]. We conclude that the presence of antinuclear autoantibodies supports the autoimmune phenotype of CBD in mice.

In conclusion, our data provide evidence for the involvement of DCs and Th17 cells or Th17.1 cells in the pathogenesis of CBD, in particular in the induction of granulomas and TLSs. The crucial role of DCs and Th17 cells may have consequences for the development of therapeutic strategies, and provide a more specific and effective target in the immunosuppressive treatment of CBD.

## Data Availability

There are no publicly archived datasets analysed in this study.

## Supplementary Materials

Supplementary 1 Supplementary Table: antibodies for FACS.

Supplementary 2 Supplementary Figure 1: weight curve of beryllium-exposed mice. Weight curve showing the gradual increase over time in the weight of mice treated with 180 *μ*g BeO or Be for 3 weeks, 3 times per week.

Supplementary 3 Supplementary Figure 2: total BAL fluid cells over time of Be-exposed C3H mice. Total BAL cell numbers shown from mice treated with a low dose (18 *μ*g) or a high dose (180 *μ*g) of AlBe, BeO, and Be, as indicated, for 3 weeks 3 times per week. Mice were sacrificed at 6 or 8 weeks after the initial exposure. The results shown are expressed as the means ± SEM and represent one out of two independent experiments with 3-6 mice per group. Mann-Whitney *U* test: ^∗^*p* < 0.05.

Supplementary 4 Supplementary Figure 3: gating strategy of BAL fluid cells. Threshold was set to exclude small BeO particles and debris. Next, based on granulation SSC and FSC, granulocytes, lymphocyte gate, monocyte gate, and row macrophages were gated. The row macrophages were subdivided in highly autofluorescent (FITC) CD11c-positive alveolar macrophages and debris including monocytes. The lymphocyte gate was subdivided into MHC class II-positive lymphocytes which were subdivided into CD11c-positive DCs and CD11c-negative B cells. DCs were subdivided into CD11b-positive cDCs, moDCs, and CD103-positive cDCs and MHC-negative CD3^+^ T cells. T cells were subdivided into CD4- and CD8-positive cells and remaining debris including BeO particles. The monocyte gate showed inflammatory monocytes and steady-state monocytes.

Supplementary 5 Supplementary Figure 4: gating strategy of lung-draining MLN cells. Flow cytometric analysis revealed that i.t. injection of 50 ng DT-depleted CD11c^+^MHCII^+^ DCs in the lung-draining MLNs of naive CD11c-DTR C3H/Hej x BALB/c (F1) mice but not of WT control littermates.

Supplementary 6 Supplementary Figure 5: BAL CD4 cytokine profiles of WT and IL-17R KO CBD mice. Quantification of the frequencies of CD4^+^ T cells that are positive for the indicated cytokines, as determined by intracellular flow cytometric analysis of BAL cells. The results shown are expressed as the means ± SEM and represent one out of two independent experiments with 4-7 mice per group. Mann-Whitney *U* test: ^∗^*p* < 0.05.
