# Supplementary material for: Involvement of Dendritic Cells and Th17 Cells in Induced Tertiary Lymphoid Structures in a Chronic Beryllium Disease Mouse Model
Source: Mediators Inflamm. 2021 May 6;2021:8845966. doi: 10.1155/2021/8845966 (PMC8123089; doi:10.1155/2021/8845966)
Supplement: Supplementary 1 — Supplementary Table: antibodies for FACS. [file 8845966.f1.docx]

Antibodies for FACS

|  |  |  |  |  |  |
| --- | --- | --- | --- | --- | --- |
| Antibody binding molecule | clone | Fluorochrome | Manufacturer |  |  |
|  | 2.4G2 |  |  |  |  |
| B220 | RA3-6B2 | AF700 | eBioscience |  |  |
| CD11b | M1/70 | PercpCy5.5 | BD |  |  |
| CD11c | N418 | PTxR | Caltag |  |  |
| CD11c | N418 | PeCy7 | BD |  |  |
| CD138 | 281-2 | APC | BD |  |  |
| CD19 | 1D3 | AF700 | eBioscience |  |  |
| CD19 | 1D3 | PercpCy5.5 | eBioscience |  |  |
| CD3 | 145-2c11 | APC | eBioscience |  |  |
| CD3 | 145-2c11 | PTR | BD |  |  |
| CD4 | RM4-5 | FITC | eBioscience |  |  |
| CD4 | RM4-5 | AF700 | eBioscience |  |  |
| CD40 | 1C10 | PE | eBioscience |  |  |
| CD64 | X54-5/7.1 | AF647 | BD |  |  |
| CD8 | 53-6.7 | PE-Cy7 | eBioscience |  |  |
| CD8 | 53-6.7 | PE | eBioscience |  |  |
| CD86 | GL1 | APC | eBioscience |  |  |
| CD95 | Jo2 | PE | BD |  |  |
| F480 | BM8 | APC AF750 | eBioscience |  |  |
| IgD | 11-26c.2a | Fitc | BD |  |  |
| IgM |  | PerCpCy5.5 |  |  |  |
| Ly6C | HK1,4 | ef450 | eBioscience |  |  |
| Ly6G | 1A8 | PE | BD |  |  |
| MHCII | I-Ad/I-Ed | FITC | eBioscience |  |  |
| PNA |  | bio |  |  |  |
| SiglecF | E50-2440 | PeCF594 | BD |  |  |
| IL-4 | 11B11 | PeCy7 | BD |  |  |
| IFNg | XMG1.2 | PerCPcy5.5 | eBioscience |  |  |
| IL-17 | TC11-18H10.1 | AF700 | BD |  |  |
| IL-13 | eBio13A | PB (v450) | eBioscience |  |  |
