# Supplementary figures and images for: Involvement of Dendritic Cells and Th17 Cells in Induced Tertiary Lymphoid Structures in a Chronic Beryllium Disease Mouse Model
Source: Mediators Inflamm. 2021 May 6;2021:8845966. doi: 10.1155/2021/8845966 (PMC8123089; doi:10.1155/2021/8845966)

Supplementary figure 1

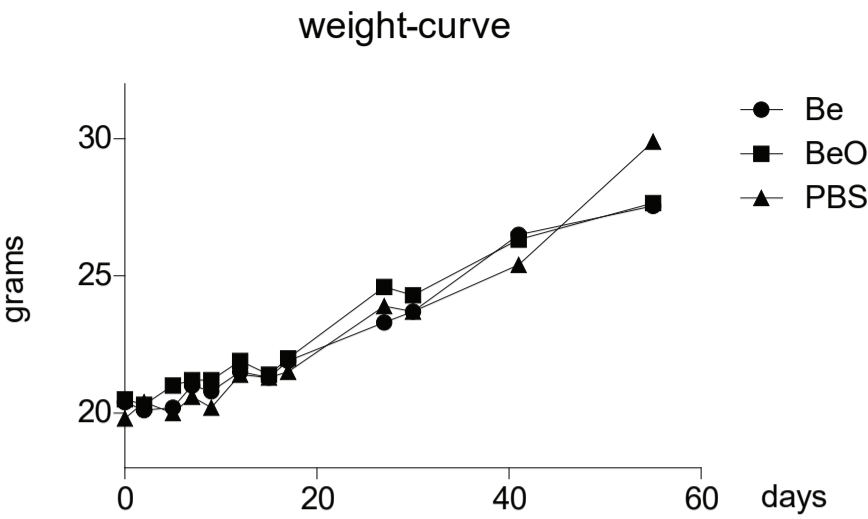

Supplement: Supplementary 2 — Supplementary Figure 1: weight curve of beryllium-exposed mice. Weight curve showing the gradual increase over time in the weight of mice treated with 180 μg BeO or Be for 3 weeks, 3 times per week. [file 8845966.f2.pdf]

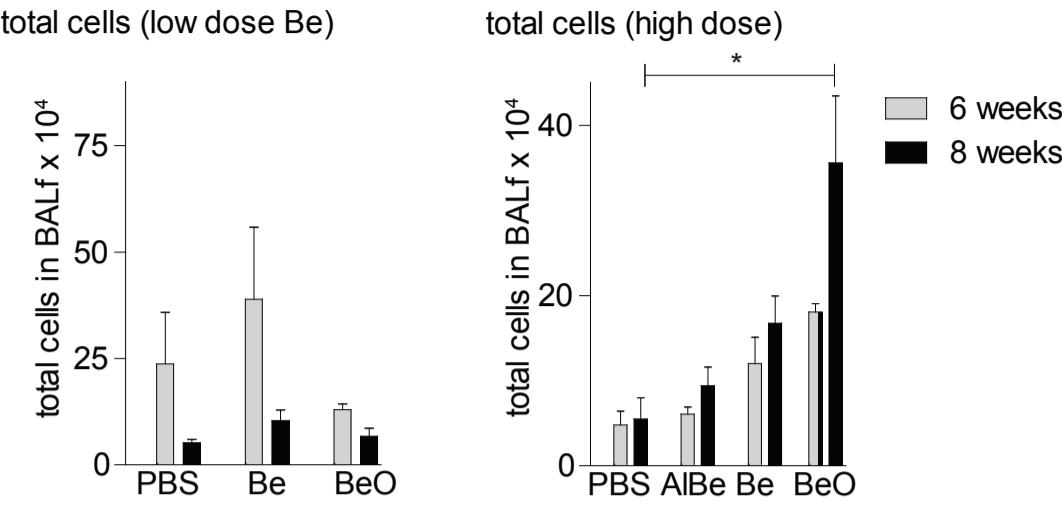

Supplement: Supplementary 3 — Supplementary Figure 2: total BAL fluid cells over time of Be-exposed C3H mice. Total BAL cell numbers shown from mice treated with a low dose (18 μg) or a high dose (180 μg) of AlBe, BeO, and Be, as indicated, for 3 weeks 3 times per week. Mice were sacrificed at 6 or 8 weeks after the initial exposure. The results shown are expressed as the means ± SEM and represent one out of two independent experiments with 3-6 mice per group. Mann-Whitney U test: ∗p < 0.05. [file 8845966.f3.pdf]

Supplementary Figure 3

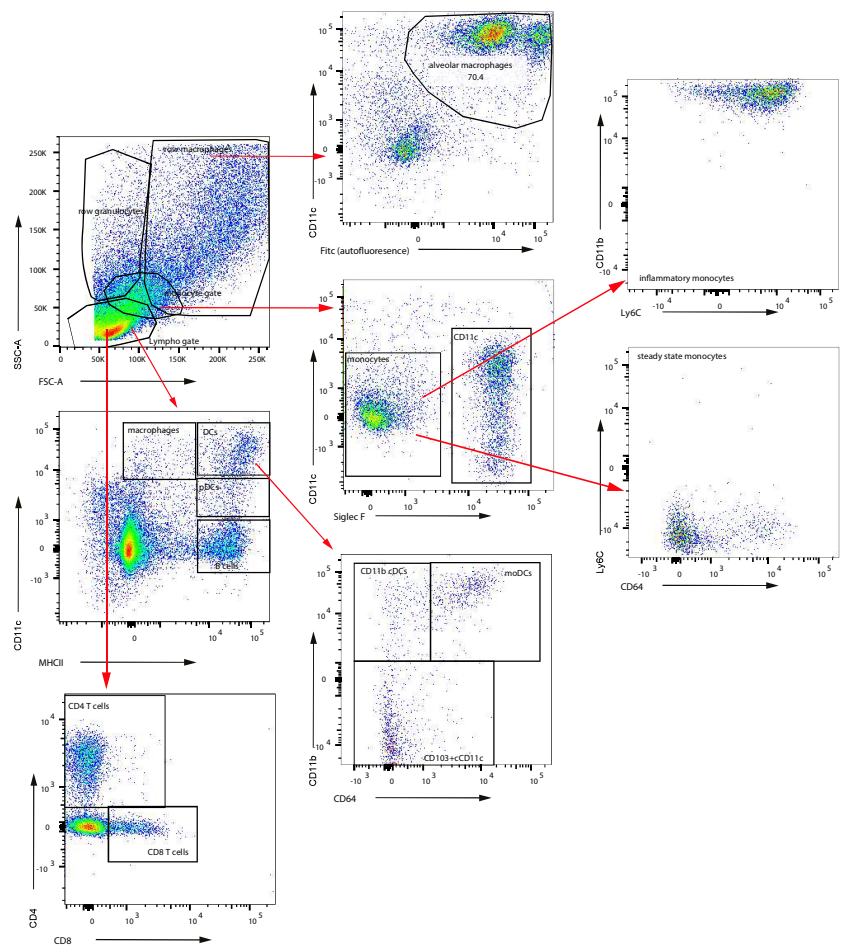

Supplement: Supplementary 4 — Supplementary Figure 3: gating strategy of BAL fluid cells. Threshold was set to exclude small BeO particles and debris. Next, based on granulation SSC and FSC, granulocytes, lymphocyte gate, monocyte gate, and row macrophages were gated. The row macrophages were subdivided in highly autofluorescent (FITC) CD11c-positive alveolar macrophages and debris including monocytes. The lymphocyte gate was subdivided into MHC class II-positive lymphocytes which were subdivided into CD11c-positive DCs and CD11c-negative B cells. DCs were subdivided into CD11b-positive cDCs, moDCs, and CD103-positive cDCs and MHC-negative CD3+ T cells. T cells were subdivided into CD4- and CD8-positive cells and remaining debris including BeO particles. The monocyte gate showed inflammatory monocytes and steady-state monocytes. [file 8845966.f4.pdf]

## Supplementary figure 4

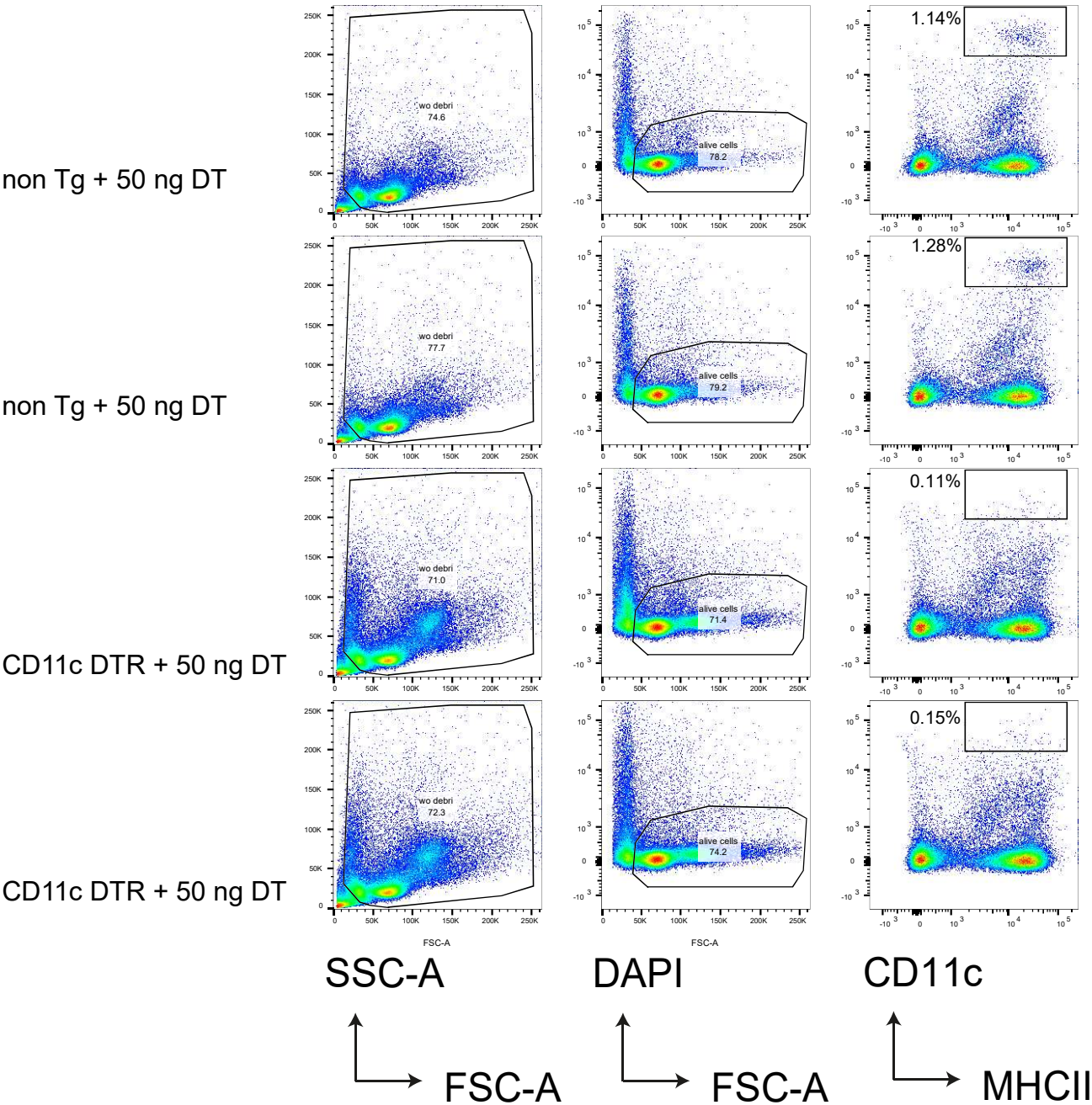

Supplement: Supplementary 5 — Supplementary Figure 4: gating strategy of lung-draining MLN cells. Flow cytometric analysis revealed that i.t. injection of 50 ng DT-depleted CD11c+MHCII+ DCs in the lung-draining MLNs of naive CD11c-DTR C3H/Hej x BALB/c (F1) mice but not of WT control littermates. [file 8845966.f5.pdf]

Supplementary figure 5

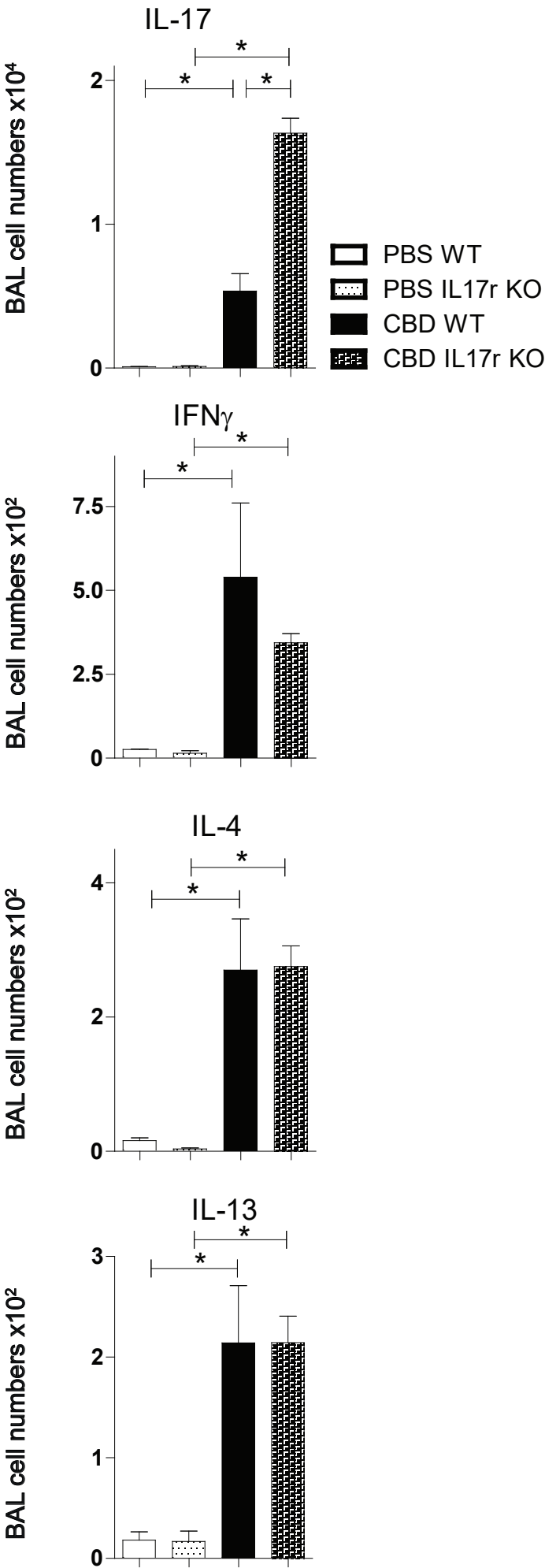

Supplement: Supplementary 6 — Supplementary Figure 5: BAL CD4 cytokine profiles of WT and IL-17R KO CBD mice. Quantification of the frequencies of CD4+ T cells that are positive for the indicated cytokines, as determined by intracellular flow cytometric analysis of BAL cells. The results shown are expressed as the means ± SEM and represent one out of two independent experiments with 4-7 mice per group. Mann-Whitney U test: ∗p < 0.05. [file 8845966.f6.pdf]
